# 糖化血红蛋白参考方法的建立及国际比对

**DOI:** 10.3724/SP.J.1123.2025.03017

**Published:** 2026-03-08

**Authors:** Mo WANG, Shunli ZHANG, Rui ZHANG, Yichuan SONG, Jie SHI, Qiaozhen XUE, Yanwei HU

**Affiliations:** 1.首都医科大学附属北京朝阳医院，北京 100020; 1. Beijing Chao-Yang Hospital，Capital Medical University，Beijing 100020，China; 2.北京市临床检验中心，北京 100020; 2. Beijing Center for Clinical Laboratory，Beijing 100020，China

**Keywords:** 糖化血红蛋白, 液相色谱-串联质谱, 国际比对, glycated hemoglobin, liquid chromatography-tandem mass spectrometry （LC-MS/MS）, international comparison

## Abstract

为促进北京地区范围内医学实验室糖化血红蛋白检测结果的标准化和一致化，北京市临床检验中心建立了糖化血红蛋白的参考方法，并参加了欧洲糖化血红蛋白参考实验室网络组织的参考实验室糖化血红蛋白A1c（HbA1c）国际比对计划以验证该方法的准确性。样本采用谷氨酸内切蛋白酶进行消化，采用高效液相色谱-串联质谱法（HPLC-MS/MS），以含0.1%甲酸的甲醇水溶液为流动相，梯度洗脱，在电喷雾离子源多反应监测模式下，对HbA1c国际样本进行检测。糖化与非糖化六肽均在3 min内出峰，标准曲线的线性相关系数为0.999 6～0.999 8，批内变异系数为0.35%～2.20%，总变异系数为0.83%～2.39%。国际临床化学和实验室医学联合会（IFCC）组织方将单个实验室结果与总体中位数之间的差异与总体中位数进行对比，并根据线性关系计算比例偏移（斜率）和系统偏移（截距），用斜率和截距共同计算得出实验室的综合统计的检验值，2018-2024年，本实验室检测结果的比例偏移范围为-0.009～0.021，系统偏移范围为-0.69～1.27，综合统计检验结果最大值为3.0，最小值为0.1。结果表明所建立的HbA1c参考方法性能良好，2018-2024年IFCC国际比对成绩满意。

## Abstract (en)

To promote the standardization and harmonization of glycated hemoglobin A1c （HbA1c） testing results in medical laboratories across Beijing， we established a reference method for HbA1c. This method participated in the International Comparison Program for HbA1c Reference Laboratories organized by the European Reference Laboratory Network for HbA1c to validate its accuracy. The experimental samples consisted of HbA1c network reference laboratory proficiency testing samples imported annually from the Netherlands， including six calibrators （A-F）， ten intercomparison samples， several quality control materials， and additional auxiliary samples with International Federation of Clinical Chemistry and Laboratory Medicine （IFCC）-assigned values. The pre-treatment process involved digesting samples with protein endopeptidase Glu-C. The experimental samples were removed from the -80 ℃ freezer and allowed to reach room temperature. Subsequently， 50 μL of Glu-C （mass concentration： 200 μg/mL） was added to each sample vial at a ratio of total hemoglobin to enzyme of 1 mg∶0.01 mg. The volume was then adjusted to a final volume of 500 μL with ammonium acetate solution （50 mmol/L， pH 4.3）. After thorough mixing， the samples were incubated at 37 ℃ for 18-20 h. Using mobile phases of methanol and 0.1% formic acid aqueous solution under gradient elution conditions， the detection of HbA1c in international samples was performed via high performance liquid chromatography-tandem mass spectrometry （HPLC-MS/MS）. A gradient elution mode was employed for liquid chromatography separation using a Shimadzu C18 column （50 mm×3 mm， 2.2 μm）. The injector temperature was maintained at 4-8 ℃， with a column temperature of 30 ℃. The flow rate was kept constant at 0.6 mL/min， with an injection volume of 5 μL and a total run time of 8 min. The MS detection was performed using electrospray ionization （ESI） in positive ion mode with multiple reaction monitoring （MRM）. The monitored ion pairs for the precursor and product ions of non-glycated and glycated N-terminal hexapeptides are *m/z* 348.2/237.2 and *m/z* 429.2/245.2， respectively. The linearity was evaluated by performing regression analysis. The HbA1c （mmol/mol） quantification in unknown samples was achieved by performing linear regression using Analyst 1.6.2 software. Peak areas were integrated， with the abscissa （X-axis） representing the known concentration ratio of HbA1c to hemoglobin A0 （HbA0）， and the ordinate （Y-axis） representing the average peak area ratio of glycated to non-glycated hexapeptides. Both glycated and non-glycated hexapeptides eluted within 3 min， with linear correlation coefficients ranging from 0.999 6 to 0.999 8. The quality control （QC） materials （≥3 types） annually provided by the IFCC were analyzed. Since each QC material in every shipment typically consists of two replicated vials， enzymatic digestion was performed in two separate batches. For each batch， five parallel samples were processed， followed by triplicate injections of each sample. Intra-assay and total coefficients of variation （CV） were calculated. The intra-assay coefficients of variation （CVs） were 0.35%-2.20%， and the total CVs were 0.83%-2.39%. From 2018 to 2024， IFCC compared individual laboratory results with the overall median， calculating proportional bias （slope） and systematic bias （intercept） based on linear regression. Our laboratory’s combined statistical test value （computed from slope and intercept） ranged from 0.1 to 3.0. The systematic bias ranged from -0.69 to 1.27， and the proportional bias ranged from -0.009 to 0.021. The residual values from all testing points in the 2024 proficiency testing （PT） program were evenly distributed around zero. Furthermore， the magnitude of residual deviations from zero was relatively small compared to those observed across the 15 participating laboratories， demonstrating satisfactory consistency in our laboratory’s analytical results. The established HbA1c reference method demonstrated robust performance， achieving satisfactory results in IFCC international comparisons from 2018 to 2024**.**

糖化血红蛋白是血红蛋白与葡萄糖经非酶促反应结合形成的产物，其中糖化血红蛋白A1c（glycated hemoglobin A1c，HbA1c）是糖化血红蛋白中最主要的亚型，反映过去2~3个月的平均血糖水平，是糖尿病患者血糖控制的重要标志物^［1-3］^，其水平升高提示糖尿病并发症风险增加^［4］^，因此HbA1c测量结果的标准化十分重要^［5］^。为保证糖化血红蛋白检测结果的准确性、一致性和可比性，不同国家或组织规定了用于比较和校准不同检测方法或实验室间检测结果的特定方法或标准，例如美国的国家糖化血红蛋白标准化计划（National Glycohemoglobin Standardization Program，NGSP）^［6］^、日本的日本糖尿病学会/日本临床化学学会（JDS/JSCC）的基于高效液相色谱（HPLC）原理的方法^［7］^和瑞典基于阳离子交换层析的Mono-S方法^［8］^。

为建立基于计量可追溯性的参考系统，国际临床化学和实验室医学联合会（International Federation of Clinical Chemistry and Laboratory Medicine，IFCC）于1995年启动了HbA1c标准化计划，并逐步建立了全球参考实验室网络系统^［9，10］^，它为系统内的参考实验室提供标准化的检测方法与参考物质，通过全球多个参考实验室间的协作与数据共享，旨在建立统一、准确的糖化血红蛋白测量标准，确保各实验室检测结果的准确性与可比性。2002年IFCC正式公布了HbA1c检测主要的参考方法为高效液相色谱-串联质谱法（HPLC-MS/MS）与高效液相色谱-毛细管电泳法（HPLC-CE）^［11，12］^。HPLC-MS/MS结合了HPLC的高效分离能力与MS的高灵敏度、高特异性检测优势，能精确测定糖化血红蛋白各组分的含量。

北京市临床检验中心为北京市临床实验室提供室间质量评价服务，为了提高北京地区医学实验室对HbA1c检测的标准化和一致化水平，在IFCC推荐方法的基础上，我们结合谷氨酸内切蛋白酶（Glu-C）酶解策略，即通过Glu-C将HbA0和HbA1c酶解为非糖化六肽和糖化六肽后，优化色谱-质谱条件，实现了非糖化六肽和糖化六肽在HPLC-MS/MS上的高效分离与定量，建立了HbA1c的参考方法，并于2014年加入全球参考实验室网络系统^［13］^，成为16个参考实验室之一，2020年通过了能力验证提供者（ISO/IEC 17043）认可^［14］^。本文通过介绍该中心优化的基于HPLC-MS/MS检测HbA1c的参考方法及对2018-2024年参与国际比对的数据进行汇总分析，以评估该方法对HbA1c的检测能力。

## 1 实验部分

### 1.1 仪器与试剂

Nexera-XR液相色谱（日本岛津公司）-Q-Trap 5500串联质谱仪（美国AB Sciex公司），PB-10酸度计（德国赛多利斯公司），H17.5R离心机（中国卢湘仪公司），BCE55I-1OCN天平（德国赛多利斯公司），X-tra300超声波清洗器（德国艾尔玛公司）和移液器（德国艾本德公司）。甲醇（HPLC级）、乙酸（纯度≥99%）、乙酸铵（纯度≥98%）购自美国Sigma公司，Glu-C为测序等级，购自罗氏诊断，氯化钠为分析纯，购自北京化工有限公司。

### 1.2 实验样本

每年自荷兰进口的HbA1c网络参考实验室比对样本，主要包含6种校准品（A~F）、10支比对样本、数量不同的质控品及其他辅助IFCC确定靶值的样品，所有样本均为经过IFCC前处理后的溶血产物，总血红蛋白含量均为1 mg。除2021年因疫情原因未参与国际比对，其余参与年份的具体样本名称及种类见表1。

**表 1:** 2018-2024年IFCC糖化血红蛋白国际比对样本

Year	Study	Calibrators	Intercomparison samples	Controls	Other controls
2018	Chicago	pcal 2014-A， B， C， D， E， F	Chicago 01-Chicago 10	LTCC-1， LTPC-1， Milano 8， Shanghai 1	CalCheck 02 （2011-E）
2019	Barcelona	pcal 2016-A， B， C， D， E， F	Barcelona 01-Barcelona 10	LTCC-1， LTPC-1， Chicago 1， Shanghai 8	pcal 2012-D， pcal 2019-A， B，C， D， E， F
2020	Seoul	pcal 2016-A， B， C， D， E， F	Seoul 01-Seoul 10	LTCC-1， LTPC-1， Chicago 1， Shanghai 8	pcal 2019-B
2022	Kotten	pcal 2019-A， B， C， D， E，F	Kotten 01-Kotten 10	LTCC-1， LTPC-1， Seoul 8， Chicago 1	LTCC-2
2023	Rome	pcal 2019-A， B， C， D， E， F	Rome 01- Rome 10	LTPC-1， Seoul 8， Kotten 9	LTCC-1， LTCC-2， pcal 2023-A， B， C， D， E， F
2024	Dubai	pcal 2023-A， B， C， D， E， F	Dubai 01-Dubai 10	LTPC-1， LTPC-2， Kotten 9， Chicago 8	LTCC-1， LTCC-2

IFCC： International Federation of Clinical Chemistry and Laboratory Medicine.

### 1.3 实验方法

#### 1.3.1 酶解

按照IFCC的参考测量方法样本前处理步骤^［11］^，从-80 ℃冰箱取出待测国际比对溶血产物样本，恢复至室温后，按照总血红蛋白∶酶为1 mg∶0.01 mg的比例，向每只样本瓶中加入50 μL Glu-C（质量浓度200 μg/mL），最后，补足乙酸铵溶液（50 mmol/L，pH 4.3）至终体积500 μL，混匀后，置于37 ℃孵育18～20 h。孵育结束后离心，取上清液5 μL，加500 μL甲醇-水（1∶1，体积比），待检测。

由于国际比对样本为溶血产物，对于新鲜全血样本HbA1c的检测，需首先将其处理为溶血产物，再进行上述酶解过程。酶解前全血样本的处理步骤如下：取2 mL新鲜全血，在2 000 g离心力下离心10 min，离心后弃去上层血浆，用10 mL生理盐水洗涤两次，离心后弃去上清液，加入10 mL 0.15 mmol/L的NaCl溶液，于37 ℃孵育4 h，然后在2 000 g离心力下离心10 min，弃去上清液，加入1 mL去离子水，即为溶血产物，为计算出酶解所需Glu-C的量，最后对其进行总血红蛋白水平的检测，并按照前述步骤进行酶解。

#### 1.3.2 HPLC-MS/MS检测

HPLC的分离采用梯度洗脱的方式，色谱柱为C18色谱柱（50 mm×3 mm，2.2 μm，岛津公司），进样器温度控制在4~8 ℃，柱温30 ℃，流速：0.6 mL/min。流动相A相：0.1%甲酸水溶液，B相：甲醇，流速保持在0.6 mL/min，进样体积5 μL，检测时间8 min。梯度洗脱程序：0～0.5 min，5%B；0.5～1.5 min，5%B～60%B；1.5～2.0 min，60%B～100%B；2.0～4.5 min，100%B；4.5～8 min，100%B～5%B。

质谱检测采用电喷雾离子源（electrospray ionization，ESI）正离子、多反应监测（multiple reaction monitor，MRM）模式进行定量检测，离子源气帘气241.325 kPa，电压5 500 V，温度500 ℃，雾化气379.225 kPa，辅助加热气344.750 kPa，去簇电压80 V，碰撞室入口电压10 V，出口电压16 V。非糖化和糖化六肽的母离子和子离子的监测离子对分别为*m/z* 348.2/237.2和*m/z* 429.2/245.2，对应的碰撞能量分别为25 V和30 V。

#### 1.3.3 数据分析

采用Analyst 1.6.2软件，峰面积经积分处理，横坐标为IFCC提供的HbA1c与血红蛋白A0（hemoglobin A0，HbA0）的浓度比，纵坐标为糖化与非糖化六肽的平均峰面积比值，做线性回归，通过线性公式，定量未知样品中的HbA1c（mmol/mol）。

#### 1.3.4 扩展不确定度的计算

根据《JCGM 100： Evaluation of Measurement Data-Guide to the Expression of Uncertainty in Measurement》^［15］^，对3个不同水平的样本，通过整合校准品的不确定度^［16］^、方法的精密度和液体转移等引起的不确定度来估算扩展不确定度。

## 2 结果与讨论

### 2.1 色谱条件及质谱条件优化

IFCC参考方法的核心理念是通过高特异性的酶解-HPLC-MS/MS或HPLC-CE，分离并定量β链N末端的糖化与非糖化六肽，从而避免传统离子交换法因血红蛋白变异体（如HbS、HbC）或干扰物质（如高浓度葡萄糖）导致的误差^［17-19］^。

等度洗脱的优势是色谱峰出峰时间稳定，不需要平衡，因此检测时间短，然而，我们尝试使用等度洗脱的方法，无论如何调整流动相的条件，非糖化六肽与糖化六肽均无法呈现比较满意的色谱峰，因此我们沿用梯度洗脱的方法，调整流动相的比例和pH值，最终使非糖化和糖化六肽在3 min内洗脱出峰（图1），整体检测时间为8 min，且出峰时间稳定，峰形满意，是目前参考方法中出峰最快的方法^［11，12，20，21］^。

**图1:**
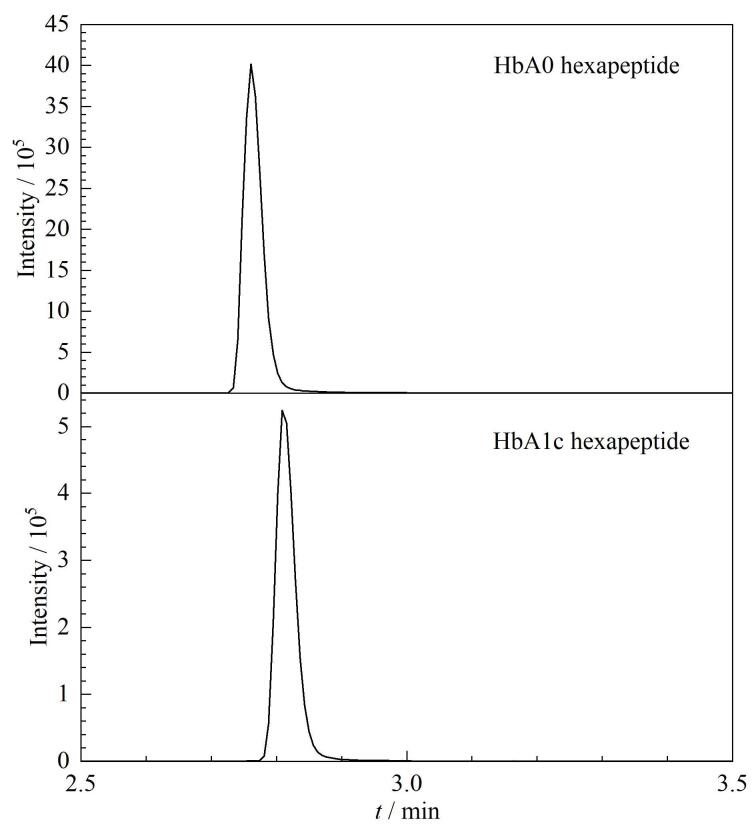
采用梯度洗脱在MRM监测模式下六肽的色谱图

根据IFCC参考测量程序^［11］^，HbA0和HbA1c经过酶消化处理后，我们曾对比了单离子监测扫描（single ion monitoring，SIM）和MRM的方法，使用的母离子和子离子分别为*m/z* 348.2/110.0及*m/z* 429.2/245.2，发现MRM较IFCC推荐的SIM模式精密度更优^［22］^。在方法的后续运行过程中，我们采用MRM模式，进一步对产生的非糖化和糖化六肽进行母离子和子离子扫描。结果显示，对于糖化六肽，信号响应最高的母离子和子离子依然为*m/z* 429.2和*m/z* 245.2，而对于非糖化六肽，子离子扫描发现*m/z* 348的母离子断裂后，灵敏度最高的子离子为*m/z* 237.2，并且通过后续研究，发现该对离子对较348.2/110.0响应更加稳定，其断裂位点分别为N端第2、3氨基酸残基之间和第4、5氨基酸残基之间，与国家卫生健康委临检中心的检测结果^［23］^一致。

人全血样本基质复杂，但前处理步骤相对简单，蛋白或肽段未经过纯化，为避免质谱仪器污染，有研究采用液相色谱分离，收集目标肽段后，再进入质谱检测^［21］^。本研究则通过切换质谱进样阀，仅使2.0~3.0 min的HPLC流出液进入质谱仪，其余时间均切换至废液排出，避免了先采用液相色谱仪富集肽段，再转移至液相色谱-串联质谱仪检测的步骤。

### 2.2 线性关系和精密度

在HPLC-MS/MS检测中，更换监测的离子对，可能因信号响应的稳定性、离子化效率的变化或受到未预期的基质干扰（如共洗脱化合物产生相同离子对）而影响方法学性能^［24］^。因此，我们重新进行了方法的性能验证，包括线性关系、精密度和准确性。准确性主要通过参与IFCC国际参考实验室间的比对来评估，结果见2.3节。

线性关系的评估方法如下：将校准品的HbA1c和HbA0的浓度比与糖化和非糖化六肽的峰面积比进行线性回归，并使用所得方程计算HbA1c浓度。表2总结了2018-2024年24批测量中，HbA1c测量值估计的斜率、截距、相关系数和标准误差。从结果可以看出，线性回归系数分布范围为0.999 6~0.999 8，标准曲线满足要求。

**表 2:** 校准品HbA1c与HbA0的浓度比与糖化和非糖化六肽的峰面积比的线性回归参数

Item	Slope （*m*）	Intercept （*b*）	Correlation coefficient （*r* ^2^）	Standard error of estimate （*S*_YX_）
Mean	1.47	-0.0001	0.9997	0.0013
Range	1.2550-1.5780	-0.0011	0.9996-0.9998	0.0001-0.0019

Peak area ratios （*Y*） were plotted against concentration ratios （*X*） and fitted to a linear regression model： *Y*=*mX*+*b.*

精密度是影响检测不确定性的因素之一。本研究通过变异系数（coefficient of variation，CV）评估精密度，方法如下：对IFCC每年邮寄的质控品（≥3种）进行检测，由于每批邮寄的每种质控品一般为2支重复，因此我们对其分两批进行消化酶解，每批做5支平行样，重复进样3次，计算批内和总变异系数，结果见表3。从结果可以看出，在靶值分布范围为31.2～81.6 mmol/mol质控品的检测中，批内变异系数最大为2.20%，最小为0.35%，总变异系数最大为2.39%，最小为0.83%。批内和总变异系数最大的样本为浓度水平较低的质控品Chicago 1。

**表 3:** HbA1c检测的批内及总变异系数

Control	Assigned value/（mmol/mol）	2018		2019		2020	
		Intra-assay CVs/%（*n*=15）	Total CV/%（*n*=30）	Intra-assay CVs/%（*n*=15）	Total CV/%（*n*=30）	Intra-assay CVs/%（*n*=15）	Total CV/%（*n*=30）
LTCC-1	59.7	0.72， 1.21	1.23	0.63， 1.20	1.41	0.49， 1.05	1.31
LTPC-1	49.9	1.10， 1.15	1.65	0.80， 0.93	1.36	0.38， 1.11	0.85
LTPC-2	49.1	/	/	/	/	/	/
Milano 8	78.8	0.98， 1.11	1.38	/	/	/	/
Shanghai 1	31.4	0.69， 0.82	0.98	/	/	/	/
Shanghai 8	78.3	/	/	0.50， 1.48	1.95	0.78， 1.29	1.94
Chicago 1	31.9	/	/	0.73， 0.93	1.41	0.80， 1.62	1.79
Chicago 8	78.6	/	/	/	/	/	/
Seoul 8	81.6	/	/	/	/	/	/
Kotten 9	31.2	/	/	/	/	/	/
Control	Assigned value/（mmol/mol）	2022		2023		2024	
		Intra-assay CVs/%（*n*=15）	Total CV/%（*n*=30）	Intra-assay CVs/%（*n*=15）	Total CV/%（*n*=30）	Intra-assay CVs/%（*n*=15）	Total CV/%（*n*=30）
LTCC-1	59.7	0.69， 0.85	1.44	0.72， 1.18	1.29	/	/
LTPC-1	49.9	0.57， 1.33	1.82	/	/	0.46， 0.88	1
LTPC-2	49.1	/	/	/	/	0.50， 1.19	1.27
Milano 8	78.8	/	/	/	/	/	/
Shanghai 1	31.4	/	/	/	/	/	/
Shanghai 8	78.3	/	/	/	/	/	/
Chicago 1	31.9	1.33， 2.20	2.39	/	/	/	/
Chicago 8	78.6	/	/	/	/	0.48， 0.66	0.97
Seoul 8	81.6	0.35， 0.63	0.83	0.63， 0.88	1.18	/	/
Kotten 9	31.2	/	/	0.56， 1.03	1.28	0.67， 0.79	1.32

### 2.3 偏倚

全球16家参考实验室参与IFCC糖化血红蛋白结果比对计划，对IFCC统一邮寄的质控品、校准品和比对样本进行检测。每年上半年和下半年各检测并汇报一次，每次5个比对样本。IFCC组织方计算出所有实验室比对样本的总体中位数，随后，将单个实验室结果与总体中位数之间的差异与总体中位数进行对比，并根据线性关系计算斜率（比例偏移，X轴）和截距（系统偏移，Y轴），将斜率与截距的偏离程度综合为一个统计量，来判断实验室是否存在整体偏差^25］^。

IFCC以HbA1c 50 mmol/mol（约为糖尿病诊断的切点值）的允许偏差1.5 mmol/mol为标准进行评估。为了确定特定实验室对给定mmol/mol HbA1c（例如50 mmol/mol糖化血红蛋白）的平均偏差，IFCC将比例偏差和系统偏差结合起来，进行组合统计检验。在1%、5%、10%或任何置信区间内，IFCC选择了两个标准确定临界值（见表4）：以2024年为例，第一个标准是1%的置信区间，为5.7（超过5.7的实验室有99%的可能性明显偏离该组的平均值）。第二个标准是10%的置信区间，为2.9（超过2.9的实验室有90%的可能性明显偏离该组的平均值）。当实验室的值高于第一个标准时，认为结果不可接受，标识在椭圆外（图2）；当高于第二个标准时，则视为警告。

**表 4:** HbA1c检测的偏移及综合统计检验值

Year	Systematic bias （abscissa）	Proportional bias （slope）	Combined statistical test outcome （critical values a，b）^*^
2018	-0.16	0.016	1.5 （10.1，5.1）
	1.27	-0.018	3.0 （10.4，5.1）
2019	-0.22	0.009	0.4 （5.8，2.8）
	0.48	-0.006	0.4 （6.1，3.0）
2020	-0.29	0.008	0.3 （5.8，2.8）
	-0.26	0.008	0.3 （6.0，3.0）
2022	0.34	-0.009	0.4 （5.8，2.9）
	0.58	-0.006	0.6 （5.9，2.9）
2023	0.30	-0.006	0.2 （5.7，2.9）
	-0.45	0.021	2.4 （5.5，2.7）
2024	-0.69	0.011	0.9 （5.7，2.9）
	-0.15	-0.002	0.1 （5.5，2.7）

* Critical value a： calculated at the 1% confidence interval； critical value b： calculated at the 10% confidence interval.

**图2:**
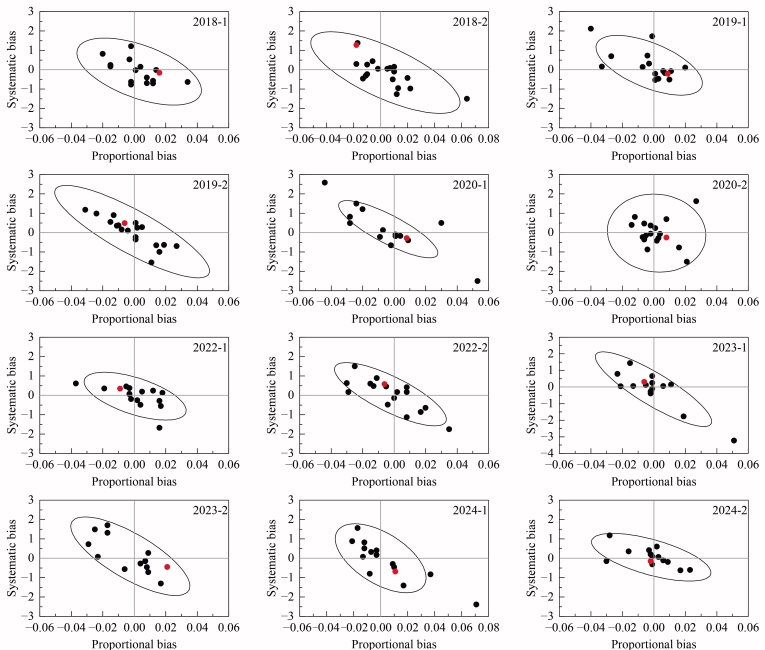
2018-2024年IFCC 16家参考实验室间比对结果（来自IFCC回报报告）

2018-2024年，本实验室检测结果的系统偏移范围为-0.69～1.27，比例偏移范围为-0.009～0.021，综合统计检验结果最大值为3.0，最小值为0.1，均未超过当年给定的第二个标准的临界值，且均在置信椭圆图范围内（红点代表本实验室结果，见图2），说明本实验室建立的HbA1c参考方法检测结果稳定，且准确度较高。

### 2.4 检测结果的一致性

检测一致性通常指结果的重复性和与理论值的吻合程度，残差（观测值与预测值或均值之间的差异）是评估检测一致性的重要工具^［26］^。在比对活动中，每个实验室实际测量的结果与靶值之间的差异即为残差。理想残差为0，残差越偏离0则该实验室的一致性就越低。

IFCC在每年HbA1c国际参考实验室比对活动结果数据分析时，将所有实验室检测结果的中位数作为该比对样本的靶值，并绘制各参加实验室比对样本检测结果的残差图，2024年Dubai样本的结果见图3，其中X轴为各个网络实验室的实验室编号，Y轴为5个比对样本检测结果的残差。北京市临床检验中心的实验室编号为Lab_24，从结果可以看出，2024年上半年及下半年比对活动的各5个检测点的残差值，在0的上下均匀分布，且在15个参与实验室中，残差偏离零点的幅度相对较小，证明本实验室的检测结果一致性满意。

**图3:**
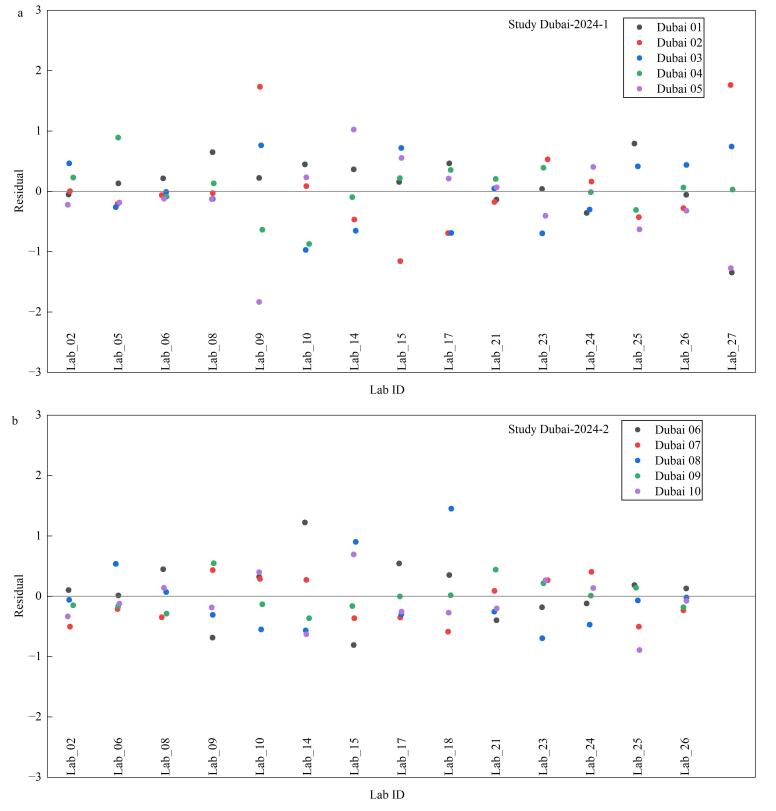
2024年IFCC比对样本结果残差图（来自IFCC回报报告）

### 2.5 检测方法的扩展不确定度

通过检测具有靶值的3个不同水平的样本，评估检测方法的扩展不确定度，结果见表5。对于浓度范围为31.2~102.5 mmol/mol的样本，标准扩展不确定度为0.96~2.33 mmol/mol。

**表 5:** HbA1检测的扩展不确定度

Samples	Target/（mmol/mol）	*U* ^*^/（mmol/mol）
Kotten 9	31.2	0.96
Seoul 8	81.6	2.33
pcal-2019-E	102.5	2.07

* Expanded uncertainty， *k*=2.

## 3 结论

糖化血红蛋白作为糖尿病诊断和长期血糖监控的核心指标，其检测结果的准确性和可比性直接关系到全球数亿糖尿病患者的临床管理^［27，28］^。然而，由于检测方法学差异、校准品特异性不足等问题，不同实验室间的结果差异曾长期存在，严重影响了临床决策的可靠性^［29］^。IFCC于2002年建立了HbA1c的参考测量方法，旨在通过标准化检测流程和高精度校准体系，实现全球实验室检测结果的溯源性和一致性^［30］^。

中国作为全球糖尿病患者最多的国家，自2015年起，逐步推进HbA1c检测标准化进程。北京市临床检验中心作为中国通过IFCC认证的3个参考实验室之一，自2014年起连续参与IFCC组织的年度国际参考实验室网络比对，比例偏移和系统偏移较低，检测准确度高，参与的比对样本的赋值结果被全球实验室采纳。同时，2018-2024年连续通过国际比对样品考核，证明了中心HbA1c参考方法定值技术能力的稳定性，确保了检测结果的国际可比性，为国内实验室提供了可溯源的标杆，为跨国多中心研究提供了数据基础。

## References

[1] Mukherjee S， Ray S K， Jadhav A A， et al . Curr Diabetes Rev， 2024， 20（7）： e251023222697 37921158 10.2174/0115733998262501231015051317

[2] Chen J， Yin D， Dou K . Cardiovasc Diabetol， 2023， 22（1）： 146 37349787 10.1186/s12933-023-01875-8 PMC10288803

[3] Gillery P . Clin Chem Lab Med， 2022， 61（5）： 861 36239682 10.1515/cclm-2022-0894

[4] Gourlay A， Sutherland C， Radley A . Prim Care Diabetes， 2024， 18（1）： 7 37925311 10.1016/j.pcd.2023.10.011

[5] Kaiser P， Reinauer H . Ger Med Sci， 2011， 9： Doc28 22135621 10.3205/000151 PMC3227124

[6] Little R R， Rohlfing C L， Wiedmeyer H M， et al . Clin Chem， 2001， 47（11）： 1985 11673367

[7] Shima K， Endo J， Oimomi M， et al . J Jpn Diabetes Soc， 1994， 37： 855

[8] Arnquist H， Wallensteen M， Jeppsson J O . Lakartidningen， 1997， 94（50）： 4789 9445960

[9] Hoelzel W， Weykamp C， Jeppsson J， et al . Clin Chem， 2004， 50（1）： 166 14709644 10.1373/clinchem.2003.024802

[10] John W G， Mosca A， Weykamp C， et al . Clin Biochem Rev， 2007， 28（4）： 163 18392123 PMC2282401

[11] Jeppsson J， Kobold U， Barr J， et al . Clin Chem Lab Med， 2002， 40（1）： 78 11916276 10.1515/CCLM.2002.016

[12] Kaiser P， Akerboom T， Molnar P， et al . Clin Chem， 2008， 54（6）： 1018 18403561 10.1373/clinchem.2007.100875

[13] Song Z X， Wang M， Zhang S L， et al . International Journal of Laboratory Medicine， 2018， 39（22）： 2729

[14] Wang M， Zhang S L， Li S， et al . Labeled Immunoassays and Clinical Medicine， 2022， 29（3）： 483

[15] Group Working 1 of the Joint Committee for Guides in Metrology （JCGM/WG 1）. JCGM 100： Evaluation of Measurement Data-Guide to the Expression of Uncertainty in Measurement. 1st Ed. （2008-09-01） ［2025-05-10］. https://www.sci.utah.edu/~kpotter/Library/Papers/jcgm:2008:EMDG/index.htmlhttps://www.sci.utah.edu/~kpotter/Library/Papers/jcgm:2008:EMDG/index.html

[16] Konnert A， Arends S， Schubert S， et al . Accredit Qual Assur， 2006， 11： 319

[17] Song Y， Xu A， Wang M， et al . Clin Chem Lab Med， 2024， 62（10）： 2082 38563053 10.1515/cclm-2024-0186

[18] Song Y， Xu A， Wang M， et al . Clin Chim Acta， 2022， 533： 168 35780822 10.1016/j.cca.2022.06.024

[19] Yadav N， Mandal A K . Clin Chim Acta， 2023， 539： 55 36476843 10.1016/j.cca.2022.11.031

[20] Kaiser P， Akerboom T， Ohlendorf R， et al . Clin Chem， 2010， 56（5）： 750 20299680 10.1373/clinchem.2009.139477

[21] Wong L， Liu H， Yong S， et al . Clin Chem， 2015， 61（2）： 435 25361947 10.1373/clinchem.2014.231340

[22] Song Z， Xie B， Ma H， et al . J Clin Lab Anal， 2016， 30（5）： 457 26510985 10.1002/jcla.21879 PMC6807171

[23] Zhang T， Zhang C， Chen W， et al . Clin Chem Lab Med， 2016， 54（4）： 569 26457776 10.1515/cclm-2015-0365

[24] Clinical and Laboratory Standards Institute . Liquid Chromatography-Mass Spectrometry Methods， 2nd ed. Wayne， Pennsylvania USA： Clinical and Laboratory Standards Institute， 2022

[25] Weykamp C， John W G， Mosca A， et al . Clin Chem， 2008， 54（2）： 240 18223132 10.1373/clinchem.2007.097402

[26] Eeckhaut A V， Lanckmans K， Sarre S， et al . J Chromatogr B， 2009， 877（23）： 2198 10.1016/j.jchromb.2009.01.003 19179125

[27] Lai L C . Malays J Pathol， 2008， 30（2）： 67 19291914

[28] Miedema K . Scand J Clin Lab Invest Suppl， 2005， 240： 61 16112961 10.1080/00365510500236143

[29] Weykamp C， John W G， Mosca A . J Diabetes Sci Technol， 2009， 3（3）： 439 20144280 10.1177/193229680900300306 PMC2769874

[30] John G， English E . Clin Chem Lab Med， 2012， 50（7）： 1243 22850056 10.1515/cclm-2011-0853

